# Lack of functional and expression homology between human and mouse aldo-keto reductase 1C enzymes: implications for modelling human cancers

**DOI:** 10.1186/1476-4598-8-121

**Published:** 2009-12-14

**Authors:** Pedro Veliça, Nicholas J Davies, Pedro P Rocha, Heinrich Schrewe, Jonathan P Ride, Chris M Bunce

**Affiliations:** 1 School of Biosciences, University of Birmingham, Edgbaston B15 2TT Birmingham, UK; 2 Department of Developmental Genetics, Max-Planck Institute for Molecular Genetics, Ihnestrasse 73, 14195 Berlin, Germany; 3 Institute of Medical Genetics, Charité-University Medicine, Berlin, Germany

## Abstract

**Background:**

Over recent years, enzymes of the aldo-keto reductase (AKR) 1C subfamily have been implicated in the progression of prostate, breast, endometrial and leukemic cancers. This is due to the ability of AKR1C enzymes to modify androgens, estrogens, progesterone and prostaglandins (PGs) in a tissue-specific manner, regulating the activity of nuclear receptors and other downstream effects. Evidence supporting a role for AKR1C enzymes in cancer derives mostly from studies with isolated primary cells from patients or immortalized cell lines. Mice are ideal organisms for *in vivo *studies, using knock-out or over-expression strains. However, the functional conservation of AKR1C enzymes between human and mice has yet to be described.

**Results:**

In this study, we have characterized and compared the four human (AKR1C1,-1C2, -1C3 and -1C4) and the eight murine (AKR1C6, -1C12, -1C13, -1C14, -1C18, -1C19, -1C20 and -1C21) isoforms in their phylogeny, substrate preference and tissue distribution. We have found divergent evolution between human and murine AKR1C enzymes that was reflected by differing substrate preference. Murine enzymes did not perform the 11β-ketoreduction of prostaglandin (PG) D_2_, an activity specific to human AKR1C3 and important in promoting leukemic cell survival. Instead, murine AKR1C6 was able to perform the 9-ketoreduction of PGE_2_, an activity absent amongst human isoforms. Nevertheless, reduction of the key steroids androstenedione, 5α-dihydrotestosterone, progesterone and estrone was found in murine isoforms. However, unlike humans, no AKR1C isoforms were detected in murine prostate, testes, uterus and haemopoietic progenitors.

**Conclusions:**

This study exposes significant lack of phylogenetic and functional homology between human and murine AKR1C enzymes. Therefore, we conclude that mice are not suitable to model the role of AKR1C in human cancers and leukemia.

## Background

Aldo-keto reductases (AKRs) are a large superfamily of ~37 kDa enzymes present in bacteria, protozoa, fungi, plants and animals [1-3]. In spite of their highly conserved (α/β)_8_-barrel structure, AKRs perform the NAD(P)H-dependent reduction of carbonyl groups in a wide variety of substrates and therefore have diverse physiological roles [3]. To date, more than 150 proteins have been assigned to the superfamily, currently divided into 15 families (AKR1-AKR15) listed at the AKR homepage http://www.med.upenn.edu/akr/[2].

**Figure 1:**
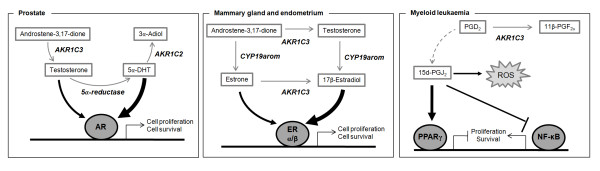
**Proposed roles of AKR1C enzymes in prostate cancer, breast cancer and myeloid leukaemia**. AR, androgen receptor. ER, estrogen receptor. CYP19arom, CYP19 aromatase. PG, prostaglandin. 15d-PGJ_2_, 15-deoxy-Δ^12,14^-PGJ_2_. ROS, reactive oxygen species. PPARγ, peroxisome proliferator-activated receptor γ. NF-κB, nuclear factor κB.

**Figure 2:**
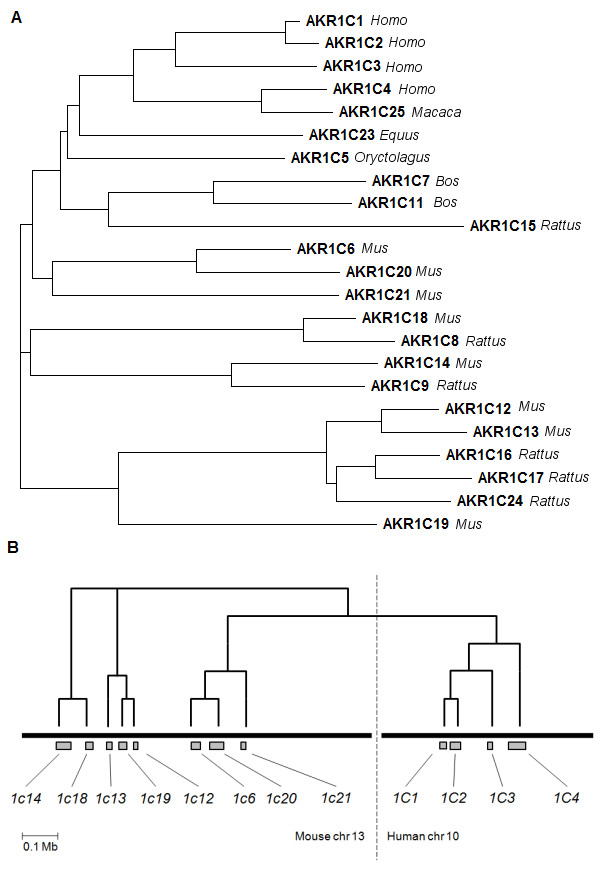
**Phylogeny of the AKR1C subfamily**. **A**. phylogenetic tree showing the relationship between the 23 known AKR1C proteins. Protein sequences available at the AKR homepage http://www.med.upenn.edu/akr/ were aligned using ClustalW and a tree generated using TreeView X. Human AKR1C isoforms cluster closely together suggesting an early common ancestor while murine isoforms have higher diversity. **B**. Position of AKR1C genes in chromosome reflects phylogenetic relationships. To scale representation of murine (chromosome 13) and human (chromosome 10) AKR1C gene clusters. Grey boxes represent individual genes.

In recent years, members of the AKR1C subfamily have been implicated in the development of human cancers due to their ability to modify steroid hormones and prostaglandins (PGs) [4-7] (Fig. 1). Four AKR1C isoforms exist in humans (AKR1C1-AKR1C4) and their respective genes are clustered on chromosome 10p14 (Fig. 2b) [5,8]. In spite of their high sequence homology (>86% amino-acid identity) human AKR1Cs have different substrate preferences and tissue distribution. All isoforms are expressed in the liver and AKR1C4 is restricted to this tissue. The remaining AKR1C1, AKR1C2 and AKR1C3 have a wider expression pattern including prostate, testes, uterus, mammary gland and haemopoietic progenitors [9-11].

In the prostate, AKR1C2 and AKR1C3 play a key role in regulating activation of the androgen receptor (AR)[5,12,13] (Fig 1). AKR1C2 inactivates the potent AR ligand 5α-dihydrotestosterone (5α-DHT) by converting it to 3α-adiol (weak AR ligand) [13,14]. On the other hand, AKR1C3 converts androstene-3,17-dione (androstenedione) to testosterone which can activate AR or be further converted to 5α-DHT by the 5α-reductase [5,15]. In fact, mRNA for AKR1C3 and AKR1C2 was found to be upregulated by 4-fold and 2-fold, respectively, in metastatic androgen-independent prostate tumours relative to its primary androgen-dependent counterparts, suggesting a role of these enzymes in the progression of the disease [16]. Furthermore, immunohistochemical analysis confirmed the upregulation of AKR1C3 protein in prostate tumours relative to normal tissue [12,16,17]. Therefore it is hypothesized that in the prostate AKR1C enzymes promote an androgenic environment beneficial for tumour progression.

AKR1C3 is also highly expressed in the mammary gland where it is thought to promote a pro-estrogenic environment (Fig. 1). In this tissue, the concerted actions of CYP19 aromatase and AKR1C3 result in the generation of estrone and estradiol and consequent activation of the estrogen receptors (ERα and ERβ) [5,18]. In addition, both AKR1C1 and AKR1C3 can convert progesterone to its inactive form 20α-hydroxyprogesterone diminishing the activation of the progesterone receptor (PR) and altering the progesterone:estrogen ratio [5,18]. As observed in prostate tumours, AKR1C3 is commonly over-expressed in malignant breast tissues [5,17]. Recently, the pro-estrogenic actions of AKR1C1 and AKR1C3 have also been implicated in the deregulated endometrium growth as both enzymes were found to be upregulated in tumour tissues relative to their adjacent normal counterparts [19].

The pro-survival properties of AKR1C enzymes are not solely related to steroid metabolism. Normal CD34^+ ^human myeloid progenitors express AKR1C1, -1C2 and -1C3 expression, the latter being largely dominant [10]. High expression of AKR1C3 was also found in primary acute myeloid leukemic cells [10]. In malignant cells, chemical inhibition or siRNA-mediated knockdown of AKR1C3 resulted in increased sensitivity to differentiation while over-expression of AKR1C3 rendered the cells more resistant [7,10,20-22]. This anti-differentiative action of AKR1C3 was shown to rely on its ability to perform the 11β-ketoreduction of prostaglandin D_2 _(PGD_2_) to 11β-PGF_2α_, also known as PGF synthase (PGFS) activity [22,23] (Fig. 1). By mediating the conversion to 11β-PGF_2α_, AKR1C3 prevents the spontaneous dehydration of PGD_2 _into the J-series prostaglandins, ultimately generating 15-deoxy-Δ^12,14^-PGJ_2 _(15d-PGJ_2_) [24]. The intracellular generation of 15d-PGJ_2 _has potent anti-neoplastic activities such as inhibition of NF-κB signalling, activation of peroxisome proliferator-activated receptor γ (PPARγ) and generation of reactive oxygen species [24,25]. Thus, in malignant cells AKR1C3 protects from the pro-differentiative effects of endogenously generated 15d-PGJ_2_.

These implications in cancer biology have sparked clinical interest in the human AKR1C enzymes. Recent efforts in developing isoform specific inhibitors reflect the potential therapeutic application of these enzymes [4,14,18,26]. Despite the large amount of evidence supporting roles for AKR1C enzymes in cancer, data regarding animal models and whole-organism *in vivo *studies are still absent. Ideally, transgenic mouse strains would inform about the physiological roles of AKR1C enzymes in breast, prostate, endometrial and haemopoietic tissues. In 2002, a genomic cluster on mouse chromosome 13 was described harbouring eight murine AKR1C genes: *Akr1c6*, *Akr1c12*, *Akr1c13*, *Akr1c14*, *Akr1c18*, *Akr1c19*, *Akr1c20 *and *Akr1c21 *[27]. The encoded proteins share between 64 and 76% sequence identity with the human isoforms. However, identification of homologues by a systematic comparison of murine and human AKR1C enzymes has yet to be performed.

In this study we characterized and compared the murine and human AKR1C enzymes, by focusing on phylogeny, substrate preference and tissue expression. We found significant evolutionary divergence between enzymes of the two species which was reflected in their divergent substrate preferences. None of the murine enzymes shared the PGD_2 _11β-ketoreductase activity of AKR1C3. However murine AKR1C6, was able to reduce PGE_2 _to PGF_2α_, an activity absent in the remaining human and mouse isoforms. Also, the preference for the steroid substrates androstenedione, progesterone, estrone and 5α-DHT revealed no clear functional homologies between human and mouse isoforms. Notably, AKR1C18 inactivated 5α-DHT to 3α-adiol with high efficiency. The divergence was also observed in tissue expression pattern, with absent or very low expression of the murine enzymes in prostate, testes and uterus. In summary, this study exposes the lack of homology between human and mouse AKR1C enzymes identifying the mouse as a poor model for studying the role of these enzymes in human cancer.

## Results

### Phylogenetic divergence between human and murine AKR1C enzymes

In order to identify evolutionary homologies between human and murine AKR1C enzymes we aligned the amino-acid sequences of the 23 known members of the subfamily and generated a phylogenetic tree (Fig. 2a). The four human isoforms are closely related (with an average of 88.3% sequence identity amongst them) indicating a recent common ancestor. In contrast, the eight murine enzymes show higher variability (average of 69.8% sequence identity amongst them) implying divergence from an earlier common ancestor. Similar diversity is also observed for the closely related rat enzymes. No obvious evolutionary parallels could be established between individual human and murine enzymes suggesting low conservation between the two species. Interestingly, organization of the AKR1C genes in both the human and murine genomic cluster strongly correlates with their phylogeny, with highly related genes sitting in close proximity on the chromosome (Fig. 2b). In summary, the phylogeny of the AKR1C subfamily suggests a divergent evolutionary path between human and murine enzymes.

### Murine and human AKR1C enzymes differ in the usage of prostaglandins and steroids as substrates

**Figure 3:**
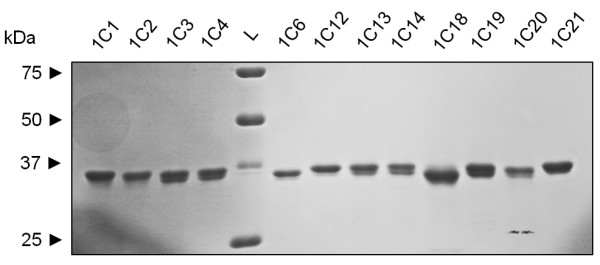
**SDS-PAGE of purified recombinant AKR1C proteins**. SDS-PAGE showing the purity of recombinant human and murine AKR1C enzymes. AKR proteins bearing a C-terminal 6× His tag were over-expressed in *E. coli *and purified in affinity column followed by FPLC. To determine purity 3 μg of protein were separated in SDS-PAGE and the gel stained with Coomassie Blue.

Even though homologies between human and murine isoforms could not be established by sequence comparison, functional homologues may occur in terms of enzymatic activity. Therefore we generated purified recombinant proteins for all human and murine AKR1C enzymes (Fig. 3) and screened for reduction of prostaglandin and steroid substrates previously implied in tumour generation or progression. All purified proteins were able to reduce the pan-substrate 9, 10-phenanthrenequinone (4 μM) with velocities ranging between 890 and 450 nmol.min^-1^.mg^-1 ^and were thus assumed to be correctly folded and functional.

Using radiolabelled substrate, we tested for the 11β-ketoreduction of PGD_2 _into 11β-PGF_2α_, an activity that amongst the human enzymes is specific to AKR1C3 and relevant to myeloid leukemic cell survival. Surprisingly, none of the murine enzymes was able to reduce PGD_2 _confirming the absence of an AKR1C3 functional homologue amongst the murine isoforms. PGF_2α_, a stereoisomer of 11β-PGF_2α_, is also a product of PGE_2 _and PGH_2 _reduction [28]. Both F_2α _isomers stimulate the FP receptor and may also play a role in disease progression [29]. Therefore, we screened for the 9-ketoreduction of PGE_2 _and the 9,11-endoperoxide reduction of PGH_2_.

Reduction of PGH_2 _was not detected with any murine AKR1C enzymes whereas murine AKR1C6 was the only isoform able to reduce PGE_2_, generating a product that co-migrates with PGF_2α _in thin layer chromatography (TLC). The reaction had low affinity (*K*_m _= 73.1 ± 19.9 μM) and low speed of turn-over (*k*_cat _0.24 ± 0.03 min^-1^), resulting in an equally low catalytic efficiency (*k*_cat_/*K*_m _= 3 min^-1^.mM^-1^).

Reduction of steroid hormones was measured using a spectophotometric assay (results are summarized in Table 1). Androstenedione (adione) was reduced by all human enzymes, with AKR1C2 and AKR1C4 being the most efficient (*k*_cat_/*K*_m _= 795 and 627 min^-1^.mM^-1^, respectively). Murine AKR1C6 and AKR1C21 were the only isoforms with detectable adione reduction activity. However, the kinetic profile of AKR1C21 did not fit a Michaelis-Menten curve, with reaction velocities increasing until ~6 μM adione (~4 min^-1^) followed by a decrease. Reduction of estrone to 17β-estradiol was not detected amongst the human enzymes. However, murine AKR1C6 and AKR1C18 were able to reduce estrone with relatively high efficiency (*k*_cat_/*K*_m _= 452 and 652 min^-1^.mM^-1^, respectively). Reduction of progesterone to its 20α-hydroxy form was detected with AKR1C4, -1C3 and -1C1, the latter being the most efficient (*k*_cat_/*K*_m _= 421 min^-1^.mM^-1^). Amongst the murine isoforms, AKR1C6, -1C18 and -1C20 were able to reduce progesterone with different efficiencies (*k*_cat_/*K*_m _= 192, 1080 and 138 min^-1^.mM^-1^, respectively). It should be noted that in spite of its relatively low affinity (*K*_*m *_= 105.7 μM) AKR1C18 showed rapid turn over of progesterone (114.12 min^-1^). Reduction of 5α-DHT was detected for all human isoforms except AKR1C3. Efficiency was highest with AKR1C4, followed by -1C2 and -1C1 (*k*_cat_/*K*_m _= 1096, 472 and 146 min^-1^.mM^-1^, respectively). Murine AKR1C18 was capable of reducing 5α-DHT with extremely high efficiency (*k*_cat_/*K*_m _= 5746 min^-1^.mM^-1^). AKR1C6 and AKR1C21 also showed activity towards 5α-DHT but, once again, the kinetic profile of AKR1C21 did not fit a Michaelis-Menten curve with reaction velocity peaking at ~15 μM (~4 min^-1^) followed by a decrease with higher substrate concentrations.

**Table 1:** Kinetic parameters for the NADPH-dependent reduction of steroid hormones by purified recombinant murine AKR isoforms.

Enzyme	*K*_m_ (μM)	*k*_cat_ (min^-1^)	*k*_cat_/*K*_m_ (min^-1^.mM^-1^)	Enzyme	*K*_m_ (μM)	*k*_cat_ (min^-1^)	*k*_cat_/*K*_m_ (min^-1^.mM^-1^)
**Adione**				**Estrone**			
AKR1C1	35.9 ± 4.4	5.24 ± 0.36	146	AKR1C1	na	na	na
AKR1C2	1.9 ± 0.7	1.51 ± 0.10	795	AKR1C2	na	na	na
AKR1C3	3.8 ± 1.0	0.79 ± 0.05	210	AKR1C3	na	na	na
AKR1C4	7.4 ± 2.1	4.64 ± 0.39	627	AKR1C4	na	na	na
							
AKR1C6	19.1 ± 4.5	10.33 ± 1.06	541	AKR1C6	11.4 ± 3.5	5.15 ± 0.58	452
AKR1C12	na	na	na	AKR1C12	na	na	na
AKR1C13	na	na	na	AKR1C13	na	na	na
AKR1C14	na	na	na	AKR1C14	na	na	na
AKR1C18	na	na	na	AKR1C18	8.9 ± 4.4	5.8 ± 0.95	652
AKR1C19	na	na	na	AKR1C19	na	na	na
AKR1C20	na	na	na	AKR1C20	na	na	na
AKR1C21	nmm	nmm	nmm	AKR1C21	na	na	na
							
**Progesterone**				**5α-DHT**			
							
AKR1C1	4.2 ± 1.0	1.77 ± 0.13	421	AKR1C1	46.9 ± 6.5	6.85 ± 0.45	146
AKR1C2	na	na	na	AKR1C2	4.3 ± 0.6	2.03 ± 0.08	472
AKR1C3	0.9 ± 0.3	0.14 ± 0.01	156	AKR1C3	na	na	na
AKR1C4	9.4 ± 3.5	0.74 ± 0.09	79	AKR1C4	6.7 ± 1.9	7.34 ± 0.66	1096
							
AKR1C6	25.1 ± 5.6	4.81 ± 0.87	192	AKR1C6	5.8 ± 2.2	0.93 ± 0.10	160
AKR1C12	na	na	na	AKR1C12	na	na	na
AKR1C13	na	na	na	AKR1C13	na	na	na
AKR1C14	na	na	na	AKR1C14	na	na	na
AKR1C18	105.7 ± 29.1	114.12 ± 19.2	1080	AKR1C18	3.9 ± 0.5	22.41 ± 0.82	5746
AKR1C19	na	na	na	AKR1C19	na	na	na
AKR1C20	3.7 ± 1.2	0.51 ± 0.05	138	AKR1C20	na	na	na
AKR1C21	na	na	na	AKR1C21	nnm	nnm	nnm

adione, androstanedione

5α-DHT, 5α-dihydrotestosterone

na, no detectable activity

nmm, data does not fit a Michaelis-Menten curve

**Figure 4:**
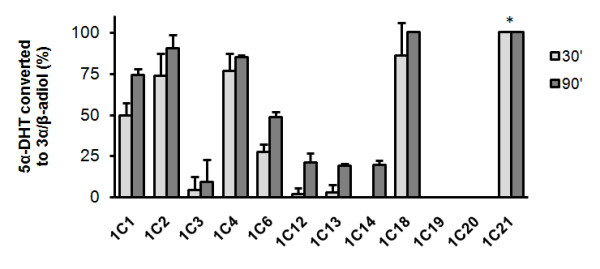
**Reduction of 5α-DHT to 3α/β-adiol by human and murine AKR1C enzymes**. Human and murine recombinant AKR1C enzymes were incubated with 25 μM 5α-DHT mixed with 1 μCi [^3^H]-5α-DHT and NADPH. At 30 and 90 minutes reactions were stopped and the steroids extracted and separated in TLC along with known standards. The radioactive traces were scanned and the percentage of 5α-DHT reduced to 3α- or 3β-adiol calculated. Bars represent average expression of three replicates and error bars represent standard deviation. * - Incubation with AKR1C21 resulted in efficient conversion to a product other than 3α- or 3β-adiol.

We have also assessed 5α-DHT reduction using radiolabelled substrate. This is a more sensitive method that allows the detection of lower activities as well as types of products formed during the reaction. Enzymes were incubated with 25 μM 5α-DHT and the percentage of substrate reduced to product was measured at 30 and 90 minutes (Fig. 4). All AKR1C isoforms except AKR1C19 and -1C20 were able to reduce 5α-DHT. In agreement with the spectrophotometric assay, AKR1C1, -1C2, -1C4, -1C18 and -1C21 were the most efficient in reducing 5α-DHT (between 50 and 100% substrate reduction at 30 minutes). AKR1C6 showed an intermediate efficiency whilst AKR1C3, -1C12, -1C13, -1C14 reduced 5α-DHT at a slow rate. Most enzymes reduced 5α-DHT to 3α-adiol with the exception of AKR1C1 that produced 3β-adiol, AKR1C4 that produced a mixture of both these stereoisomers and AKR1C21 that generated an alternative product that did not co-migrate with 3α- or 3β-adiol.

Importantly, the above analyses did not reveal any clear homologies in substrate preference between human and murine AKR1C enzymes, underlining the divergent evolutionary history of these proteins.

### Human and murine AKR1C enzymes have different tissue expression patterns

**Figure 5:**
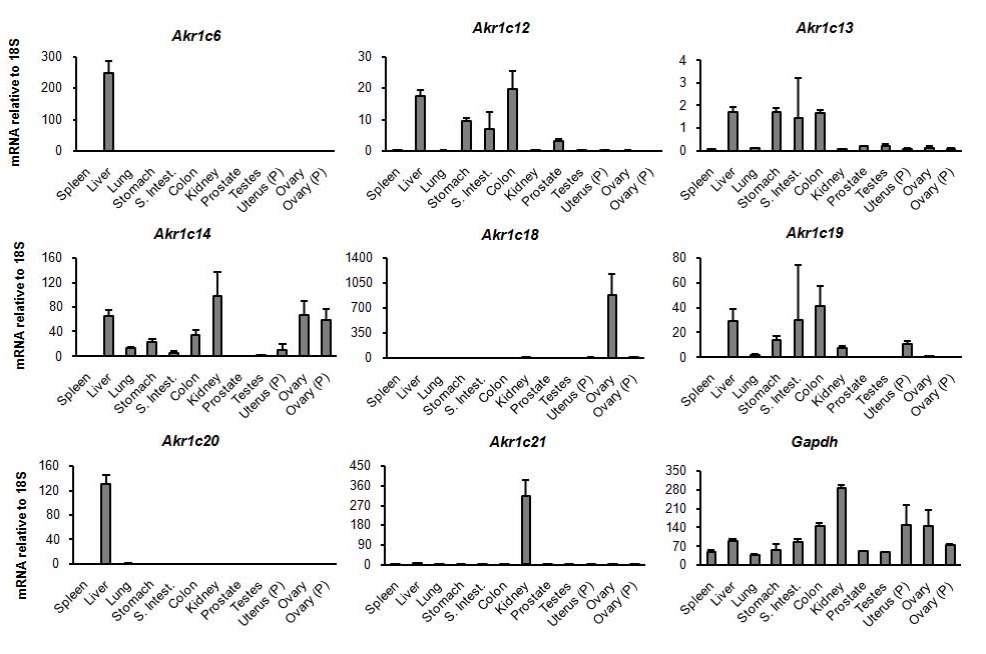
**Tissue expression of the eight murine AKR1C enzymes**. RNA was extracted from several murine tissues (in triplicate), converted to cDNA and gene expression quantified by Taqman QRT-PCR. Expression levels were normalized to *18S *expression using the following formula: (2^-(CTgene-CT18S)^)x10^6 ^and *Gapdh *expression was measured as control of cDNA quality. P - extracted from pregnant mice. Bars represent average expression of three biological replicates and error bars represent standard deviation.

Besides being expressed in the liver, human AKR1C enzymes are also found in steroid hormone-sensitive tissues such as prostate, testis, uterus and mammary gland, where they are believed to regulate steroid metabolism [9]. We measured the expression of the eight murine AKR1C genes and two house-keeping genes (*Gapdh *and *18S *ribosomal RNA) in several mouse tissues using quantitative real-time PCR (Fig. 5). Since the expression of *Gapdh *was found to be more variable amongst the analysed tissues, levels of *18S *were used as a loading control reference. Most AKR1C genes were found to be expressed in the liver, particularly *Akr1c6 *and -*1c20 *which were detected exclusively in this tissue. Tissue specificity was also observed for *Akr1c21*, detected only in the kidney. Progesterone-inactivating *Akr1c18 *was highly expressed in the ovaries of non-pregnant mice but was absent in ovaries of pregnant mice. The closely related *Akr1c12*, -*1c13 *and -*1c19 *were detected mainly in gastrointestinal tissues (stomach, small intestine and colon), though the expression levels of *Akr1c13 *were quite reduced. *Akr1c14 *showed a broader tissue distribution being detected in liver, lung, stomach, colon, kidney, uterus and ovary. Surprisingly, very low or no expression of AKR1C isoforms was detected in prostate, testes and uterus. We also screened for AKR1C expression in the mouse haemopoietic progenitor cell line HPC-7 [30,31] but none of the isoforms was detected. This result further suggests a different role of AKR1C enzymes in murine physiology.

## Discussion

The increasing interest in the role of AKR1C enzymes in cancer has brought the need for genetically modified animal models. The mouse is a powerful model organism for the study of cancer and has provided important evidence on the physiological role of many cancer-related genes. In this study we have attempted to identify homologies between human and murine AKR1C isoforms, a first step towards generating animal models. However, our results have exposed a substantial lack of homology between enzymes of the two species in terms of phylogeny, function and tissue expression.

Amino-acid sequence alignment of all known AKR1C enzymes confirmed the close evolutionary kinship of the four human AKR1C enzymes as opposed to the far more diverse murine isoforms. Previously described evolutionary homologues were also identified in the phylogram (Fig. 2a). For instance, AKR1C25, an enzyme from macaque liver, is the homologue of human AKR1C4 in substrate specificity and inhibitor sensitivity [32]. Also, murine AKR1C18 and rat AKR1C8 are known homologues for their ability to convert progesterone to 20α-hydroxyprogesterone in the ovary [33,34]. Such parallelisms were not observed between human and murine enzymes, and therefore appear to be limited to closely related species (human and macaque; rat and mouse). Overall, this analysis implies that AKR1C enzymes have diverged significantly between mice and humans since their latest common ancestor. In their enlarged array of AKR1C enzymes, mice have three isoforms more closely related to the human proteins (AKR1C6, -1C20 and -1C21), and two other subgroups that appear to have ancestral origins in the subfamily (AKR1C18 and -1C14; -1C12, -1C13 and -1C19). This phylogeny is impressively recapitulated by the position of the respective AKR1C genes in the genomic cluster (Fig. 2b), a likely record of the gene duplication history.

In this study we have also characterized murine and human AKR1C enzymes in terms of their substrate preference and tissue expression. Importantly, all enzymatic assays were performed in identical conditions in order to enhance comparability. The first striking finding is the absence of a PGD_2 _reductase amongst murine enzymes. In human malignant myeloid cells and, possibly, in haemopoietic progenitors AKR1C3 regulates cell survival and differentiation by limiting the conversion of PGD_2 _to 15d-PGJ_2_. Indeed the bone marrow, where the haemopoietic progenitors reside, was shown to be a PGD_2 _rich-environment in rodents [35]. However, the absence of an AKR1C3 homologue in mice suggests that the protection from PGD_2 _is either performed by another group of enzymes (AKR or non-AKR) or does not exist at all in murine haemopoietic progenitors. This hypothesis is further supported by the absence of AKR1C expression in the murine haemopoietic progenitor cell line HPC-7. These results demonstrate that mice can not be used to model the role of AKR1C enzymes in myeloid leukaemia.

We have also shown for the first time that AKR1C6 has PGE_2 _reductase activity. This activity is not present in the human isoforms but has been attributed to rabbit AKR1C5 [36]. Although recombinant AKR1C6 reduced PGE_2 _with poor efficiency (*k*_cat_/*K*_m _= 3 min^-1^.mM^-1^) these parameters are comparable to those of AKR1C5. Reduction of PGE_2 _can locally modify the PGE_2_/PGF_2α _signalling ratio. This is particularly important during female estrous cycle and parturition, where increased PGF_2α _levels induce degeneration of the corpus luteum and uterine contraction, respectively [37]. Also, increased PGE_2 _levels have been implicated in promoting colon cancer progression [38]. Reduction of the prostanoid precursor PGH_2 _also results in PGF_2α_. However, this activity was not detected with murine AKR1C enzymes.

The lack of parallelism was also extended to steroid turn-over. While most human AKR1C enzymes showed activity against androgens (androstenedione and 5α-DHT), only a small number of murine isoforms performed these activities. AKR1C21 and AKR1C6 were active towards androstenedione and 5α-DHT while AKR1C18 showed strong activity towards 5α-DHT only. But while the human isoforms are highly expressed in testes and prostate, AKR1C6, -1C21 and -1C18 are not, being instead restricted to liver, kidney and ovary, respectively. Therefore, despite the existence of androgen modifying AKR1C enzymes in mice, these activities are not located in the expected androgen-sensitive tissues.

Estrone reduction was not detected with any human enzyme using the spectrophotometric assay. However, previous reports have shown this activity using radiolabelled substrates with purified recombinant enzymes or cell lines over-expressing AKR1C3 [9,18]. Nevertheless, estrone reduction was easily detected with AKR1C6 and AKR1C18 despite neither enzyme is expressed in the uterus. However, AKR1C18 is highly expressed in the non-pregnant ovary where the pro-estrogenic production of estradiol might play a role.

Amongst the human enzymes, AKR1C1 showed the highest activity against progesterone as reported previously [9]. Murine AKR1C6, -1C18 and -1C20 also displayed this activity. AKR1C18 was previously shown to inactivate progesterone in endometrial and ovarian cells [33]. Expression of AKR1C18 in these tissues is suppressed during pregnancy and progesterone levels remain high. In the late stages of pregnancy AKR1C18 is re-expressed in the ovaries and uterus where it mediates the rapid inactivation of progesterone, leading to parturition [33,34]. In agreement with this, we have shown high expression of *Akr1c18 *mRNA in the ovary of pregnant mice but not in the ovaries of non-pregnant mice. AKR1C18 is also the only member of the subfamily targeted in knock-out studies [39]. Mice lacking AKR1C18 were viable but sustained high progesterone levels at the end of pregnancy resulting in delayed parturition. It should be noted that we showed here for the first time that AKR1C18 also has potent 5α-DHT and estrone reductase activity, thus contributing to a pro-estrogenic and anti-androgenic environment in the ovary. Liver-restricted AKR1C20 also showed activity against progesterone and might function as a slow inactivator of circulating progesterone. Nevertheless, the comparison revealed no clear homologies between human and murine enzymes involved in estrogen production and progesterone inactivation.

It is worth noting that AKR1C6 was able to reduce most of the substrates tested showing 3-, 17- and 20-ketoreduction activity against steroids and 9-ketoreduction against prostaglandins. Such promiscuity of this liver restricted enzyme suggests that AKR1C6 can accommodate a wide variety of substrates. Therefore, it might function as a multi-purpose detoxifying enzyme against xenobiotics. On the other hand, AKR1C12, -1C13, -1C14 and -1C19 were poorly active or inactive towards the tested substrates. Our results and previous reports have shown expression of these enzymes in the gastrointestinal tract [27,40] where they are possibly involved in reducing exogenous dietary compounds.

In this study, reduction of steroid substrates was assessed using mainly a spectrophotometric assay. This method measures the real-time consumption of NADPH in the presence of substrates and can easily be applied to a high number of enzyme/substrate combinations. However, this assay does not inform about the type of products generated in the reaction and its sensitivity is limited. Therefore, slower activities (with low *K*_*m *_and *k*_*cat*_) might be undetected. To address this we used a radiometric assay for 5α-DHT which allows for detection of modifications of the steroid substrate. In consistence with the spectrophotometric data, AKR1C1, -1C2, -1C4, -1C18 and -1C21 rapidly reduced 5α-DHT, with AKR1C6 showing lower activity (Fig. 4). However, the reduced activities of AKR1C3, -1C12, -1C13 and -1C14 against 5α-DHT were only detected using the radiometric method. This exposes the limitations of the spectrophotometric assay and alerts for a critical analysis of the presented substrate preferences since lower activities against androstenedione, progesterone and estrone might have been undetected. While most AKR1C enzymes catalyzed the reduction of 5α-DHT to 3α-adiol, incubation with AKR1C1 resulted in production of the stereoisomer 3β-adiol while AKR1C4 produced a mixture of both isomers as previously reported [15]. Confirming the divergence between human and murine AKR1C isoforms, none of the murine enzymes produced 3β-adiol. However, AKR1C21 converted 5α-DHT into an unknown product that did not co-migrate with 3α- or 3β-adiol. A non-Michaelis-Menten kinetics towards androstenedione and 5α-DHT was also observed for AKR1C21 (Table 1) underlining its unusual behaviour compared to the remaining AKR1C enzymes. These differences may relate to its ability to bind steroids differently as described previously [41]. Nevertheless, it should also be noted that some parameters of the enzymatic activities of human isoforms shown here differ from previous reports [9,12]. This is likely due to technical differences in the enzymatic assays.

This study also reveals new and interesting properties of the AKR1C subfamily. Despite being part of a highly conserved superfamily, AKR1C genes retain significant variability in gene number and sequence between species of the same class (mammalia). This may suggest that the functions of AKR1C enzymes are not strictly conserved in all mammals, instead adapting to the species evolutionary demands.

## Conclusions

Our study has demonstrated that the mouse is a poor system to model the role of AKR1C in cancer, due to the lack of functional conservation of the enzymes. Further investigations on this topic should consider animal models closer to the primates, especially when it comes to test new potential anti-cancer drugs.

## Methods

### Chemicals

NADPH, 9, 10-phenanthrenequinone (PQ), 5α-androstane-3,17-dione (androstenedione), 4-pregnene-3,20-dione (progesterone), 1,3,5(10)-estratrien-3-ol-17-one (estrone) and 5α-androstan-17β-ol-3-one (5α-dihydrotestosterone; 5α-DHT) were purchased from Sigma (Dorset, UK). Prostaglandin D_2 _(PGD_2_) and prostaglandin E_2 _(PGE_2_) were purchased from Biomol (Exeter, UK). [^3^H]-5α-DHT, [^3^H]-PGD_2 _and [^3^H]-PGE_2 _were purchased from GE Healthcare (Slough, UK). [^3^H]-3α-adiol was purchased from Perkin Elmer (Massachusetts, USA). [^3^H]-PGH_2 _was purchased from Cayman Chemical (Tallinn, Estonia).

### Multiple alignments of AKR1C proteins

AKR1C amino-acid sequences were obtained from the AKR homepage http://www.med.upenn.edu/akr/[2] and aligned using ClustalX2 http://bips.u-strasbg.fr/fr/Documentation/ClustalX/[42]. Phylogenetic tree was generated using TreeView X software http://darwin.zoology.gla.ac.uk/~rpage/treeviewx with random number generator of 111 and number of bootstrap trials of 1000.

### Production of purified recombinant AKR1C enzymes

The coding regions of human *AKR1C1, -1C2, -1C3, -1C4 *and murine *Akr1c6*, -*1c12*, -*1c13*, -*1c14*, -*1c18*, -*1c19*, -*1c20 *and -*1c21 *were amplified from several cDNA sources (tissues and cell lines) using an RT-PCR strategy. Primers introduced a 3' *Nde*I restriction site coincident with the ATG start codon and a 5' *Xho*I site before the stop codon. Coding regions were cloned into the *Nde*I and *Xho*I sites of pET28b(+) vector (Novagen, Nottingham, UK) and nucleotide sequence was confirmed by sequencing. The expression plasmid incorporates an N-terminal histidine (6 × His) tag to aid purification. For expression, the plasmid transformed into *E. coli *BL21 (DE3) (Novagen). Recombinant proteins were expressed and purified using "His-bind" resin as described previously [43]. Purity of the extracted protein was assessed by SDS-PAGE stained with Coomassie Blue. PQ turn-over was used to confirm enzymatic activity using the spectrophotometric assay described below.

### Enzymatic assays

Prostaglandin and 5α-DHT reduction was measured using a radiochemical assay. Reaction mix was composed of 150 μM NADPH, 0.2 μCi of either [^3^H]-PGD_2_, [^3^H]-PGE_2 _or [^3^H]-PGH_2 _and 35 μg/ml of recombinant enzyme in 0.2 ml of 50 mM KH_2_PO_4 _buffer (pH 6.5). Reactions were initiated upon addition of enzyme and were incubated at 37°C for 2 hours (10 minutes for PGH_2_). For determination of AKR1C6/PGE_2 _enzyme kinetics, various concentrations of cold PGE_2 _were mixed with 0.2 μCi [^3^H]-PGE_2_. For the reduction of 5α-DHT, 25 μM of cold steroid were mixed with 1 μCi [^3^H]-5α-DHT. Reactions were stopped at desired time-points by addition of methanol and initiation of the extraction protocol. PGs and steroids were extracted, separated and detected as described previously [44]. For optimal separation of 5α-DHT products, the TLC plates were developed thrice in the same solvent solution. The TLC plates were completely dried between separations.

A spectrophotometric assay was used to measure PQ, androstenedione, progesterone, estrone and 5α-DHT reduction. Reaction mix contained 150 μM NADPH, various concentrations of substrates (50 - 0.781 μM) and 15 μg/ml recombinant enzyme in 1 ml of 50 mM KH_2_PO_4 _buffer (pH 6.5). Reactions were initiated by addition of enzyme and the reaction followed at 37°C by monitoring the decrease in absorbance at 340 nm of the cofactor (ε = 6220 M^-1^.cm^-1^). For each reaction, kinetic parameters were obtained by fitting the data to a Michaelis-Menten curve, using the VisualEnzymics software (SoftZymics).

### Tissue expression analysis

Three adult male, three adult non-pregnant female and three adult pregnant female CD1 mice were sacrificed for the dissection of spleen, liver, lung, stomach, small intestine, colon, kidney, prostate, testes, uterus and ovary (in triplicate). RNA was extracted from the tissues using the RNeasy kit (Qiagen, West Sussex, UK) and cDNA produced by reverse-transcription PCR. Quantitative Real-Time (QRT)-PCR for each sample was performed in triplicate 20 μl reactions using standard cycle conditions for 44 cycles on an ABI Prism 7000 sequence detector (Applied Biosystems, Warrington, UK). Gene specific primers and TaqMan probes were designed using Primer Express software (ABI Prism) and specificity was tested using the cloned coding regions of each isoform. Sequences are shown in Table 2. TaqMan probes and QRT-PCR mix were purchased from Eurogentec (Seraing, Belgium). Gene expression in each sample was normalized to *18S *expression using the following equation: (2^-(CTgene-CT18*S*)^) × 10^6^.

**Table 2:** Sequence of primers and probes used for Taqman real-time quantitative PCR

Gene	Oligo	Sequence	Accession Number
*Gapdh*	Forward	5'-GACGGCCGCATCTTCTTGT-3'	
	Probe	JOE-5'-CAGTGCCAGCCTCGTCCCGTAGA-3'-BHQ	[EMBL:DQ403054]
	Reverse	5'-CACACCGACCTTCACCATTTT-3'	
*18S*	Forward	5'-GCCGCTAGAGGTGAAATTCTTG-3'	
	Probe	VIC-5'-TCTGGTCCGTCTTGCGCCGG-3'-Tamra	[EMBL: M27358]
	Reverse	5'-CATTCTTGGCAAATGCTTTCG-3'	
*Akr1c6*	Forward	5'-CGTCCAGAACTCGTACGG-3'	
	Probe	FAM-5'-TGCTTGGAACAGTCATTGAAGCAACTCCA-3'-Tamra	[EMBL: BC056643]
	Reverse	5'-GAGGTACAGGTCCACATAGTCCAA-3'	
*Akr1c12*	Forward	5'-TTTGTGATGTGGCCAAAAGG-3'	
	Probe	FAM-5'-AAGCCCTGCCCTAATTGCACTTCGAT-3'-Tamra	[EMBL: BC063780]
	Reverse	5'-GGCACAATCCCACGTTGAA-3'	
*Akr1c13*	Forward	5'-ATCATGGCTTCTGCCTGATGT-3'	
	Probe	FAM-5'-TGTGTGTGGACAGTGATGCTGGCAATATG-3'-Tamra	[EMBL: BC021937]
	Reverse	5'-ACAAGTCCATCCAACAGTCCATCT-3'	
*Akr1c14*	Forward	5'-CTACGGATGATTGTGAATCACCAT-3'	
	Probe	FAM-5'-TGGGCACTGGGAGCCAAACCTAAGT-3'-Tamra	[EMBL: BC013482]
	Reverse	5'-GGCACTTGCTGCTCTAACAGAA-3'	
*Akr1c18*	Forward	5'-GCCCTTGCCACGAGTTCTATTA-3'	
	Probe	FAM-5'-TTGTGTGATGCTGGACTCTCAGATGCC-3'-Tamra	[EMBL: BC034259]
	Reverse	5'-GGAGGCGGTGTGTCGAGTT-3'	
*Akr1c19*	Forward	5'-AGCAATGAGTTCCAAACAGCAA-3'	
	Probe	FAM-5'-ATGGCAACTTCATCCCTGCGCTG-3'-Tamra	[EMBL: BC087964]
	Reverse	5'-CTGGTTTGTAGGTGCCAAAGC-3'	
*Akr1c20*	Forward	5'-AACGCCTGCGCTGATTG-3'	
	Probe	FAM-5'-CCTTCGCTACCAGGTGCAACGTGG-3'-Tamra	[EMBL: BC021607]
	Reverse	5'-GAAACTCTTGGCTAGGACCACAA-3'	
*Akr1c21*	Forward	5'-CCATGGCAAAAAAATATAATCGAACT-3'	
	Probe	FAM-5'-CAGCCTTGATTGCCCTTCGCTACCA-3'-Tamra	[EMBL: BC061057]
	Reverse	5'-GACCACAATCCCACGCTGTA-3'	

## Competing interests

The authors declare that they have no competing interests.

## Authors' contributions

PV: carried out the sequence alignment of the AKR1C subfamily, cloning of the murine AKR1C isoforms, production of murine recombinant proteins, enzyme assays, tissue expression by quantitative RT-PCR and manuscript drafting. NJD and JPR: carried out the cloning and production of human AKR1C proteins and assisted in the enzyme assays. PPR and HS: carried out the extraction of mouse tissues. CMB: conceived of the study, and participated in its design and coordination and helped to draft the manuscript. All authors read and approved the final manuscript.
