# Correction: Lack of functional and expression homology between human and mouse aldo-keto reductase 1C enzymes: implications for modelling human cancers

**DOI:** 10.1186/1476-4598-9-21

**Published:** 2010-01-28

**Authors:** Pedro Veliça, Nicholas J Davies, Pedro P Rocha, Heinrich Schrewe, Jonathan P Ride, Chris M Bunce

**Affiliations:** 1 School of Biosciences, University of Birmingham, Edgbaston B15 2TT Birmingham, UK; 2 Department of Developmental Genetics, Max-Planck Institute for Molecular Genetics, Ihnestrasse 73, 14195 Berlin, Germany; 3 Institute of Medical Genetics, Charité-University Medicine, Berlin, Germany

## Correction

It has come to our attention that since publication of our article [1], in the body of the article the term androstenedione has been used in error. The experiments in this study used androstanedione as a test substrate. Androstenedione was not used in any experiments. This error does not in any way invalidate the conclusion of the paper that aldo-keto reductases of the mouse and human 1C-subfamilies lack functional and expression homology. The authors apologise for any confusion caused.
